# Isolated Native Tricuspid Valve Endocarditis in a Nonintravenous Drug User

**DOI:** 10.1155/2020/8812597

**Published:** 2020-11-22

**Authors:** Habtewold Shibru, Ermias shenkutie Greffie, Zenahbezu Abay, Oumer Abdu Muhie

**Affiliations:** ^1^Department of Internal Medicine, University of Gondar, Gondar, Ethiopia; ^2^Department of Internal Medicine, GAMBY Teaching General Hospital, Bahir Dar, Ethiopia

## Abstract

Infective endocarditis (IE) is a disease characterized by high morbidity and mortality. IE was first described in the mid-16th century. Right-sided infective endocarditis (RSIE) represents 5% to 10% of all IE episodes in adults. RSIE can be divided into three groups according to the underlying risk factors: intravenous drug users (IDUs), cardiac device carriers, and the “three noes” group (no left-sided IE, no IDUs, and no cardiac devices). Tricuspid valve endocarditis in nonintravenous drug users can occur in a variety of conditions including congenital heart disease, intracardiac devices, central venous catheters, and immunologically debilitated patients. Due to the rareness of isolated native nonrheumatic tricuspid valve endocarditis, here, we like to present an 18-year-old male from rural Ethiopia with the diagnosis of isolated native tricuspid valve endocarditis that was treated and cured.

## 1. Clinical Profile of the Patient

A previously healthy, 18-year-old male patient from Gorgora, North West Ethiopia, presented to the University of Gondar Hospital with progressive dyspnea associated with orthopnea, palpitation, bilateral leg swelling, cough productive of blood-tinged sputum, and right-sided pleuritic chest pain that lasted for a week before his visit. A week prior to the onset of the above symptoms, he had left knee joint swelling associated with high-grade fever. He noticed decreased urine amount since three days prior to admission with reddish discoloration. He had piercing injury to his right foot which formed a wound that healed by itself two months before presentation. He never had a history of intravenous drug use. He neither had a history of dental, genitourinary, or gastrointestinal procedures. He has no previous cardiac surgery.

The physical examination of this patient at presentation to the Emergency Department revealed a critically sick febrile patient in cardiopulmonary distress with low oxygen saturation. Respiratory examination showed bronchial breath sound and signs of right-side pleura effusion. Likewise, the cardiovascular examination was remarkable for raised jugular venous pulsations (JVP), displaced apical impulse, and a murmur of Tricuspid regurgitation. He also had tender hepatomegaly with a tipped spleen (3 cm below left costal margin). Musculoskeletal examination showed swollen tender right knee joint, palmar pallor, deeply dark patches on the lateral side of left foot, clubbing, and peripheral edema.

**Figure 1:**
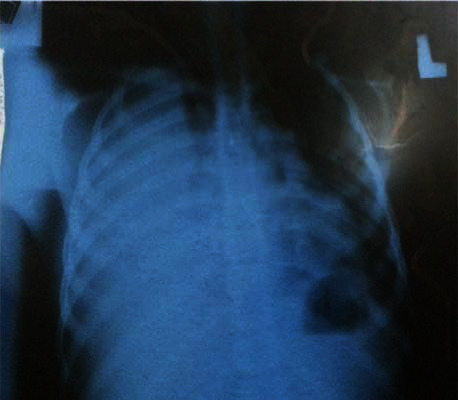
Chest roentgenography of the patient showing massive right pleural effusion.

**Figure 2:**
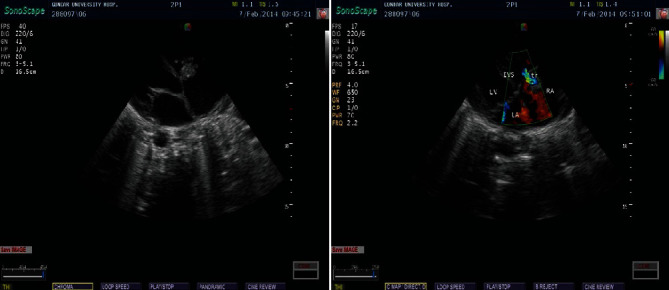
Doppler echocardiography showing an echogenic mobile focus (2.4 cm by 1.7 cm) attached to the anterior cusp of the valve (a) with moderate tricuspid regurgitation (b).

He was diagnosed with acute decompensated heart failure and severe multilobar pneumonia and was admitted to the intensive care unit and started on standard heart failure management and intravenous antibiotics. Chest radiography revealed homogenous opacity on the right hemichest. This is depicted in Figure 1. Echocardiography study showed an echogenic mobile focus attached to the anterior cusp of the tricuspid valve with moderate tricuspid regurgitation. The echocardiography result of the patient is depicted in Figure 2. Further laboratory investigations revealed leucocytosis with anemia (white cell count—32,200/*μ*l and hemoglobin—5 gm/dl), erythrocyte sedimentation rate of 159 mm/hr, elevated blood urea nitrogen, and creatinine (190 mg/dl and 1.6 mg/dl subsequently). Urine analysis showed microscopic hematuria. Rapid test for HIV infection was negative. Blood culture that was done after two doses of intravenous antibiotics showed no growth. Both pleural and joint fluid aspirate cultures showed growth of *Staphylococcus aureus* with sensitivity to several antibiotics including cloxacillin.

After the echocardiography evaluation, the patient's diagnosis was changed to tricuspid valve infective endocarditis with septic arthritis and septic pulmonary emboli. He was managed for acute heart failure and infective endocarditis with intravenous diuretics and intravenous antibiotics (ceftriaxone plus vancomycin which was later changed to cloxacillin after the antibiogram result). He also received supportive treatment with intranasal oxygen and blood transfusion. We did therapeutic pleurocentesis and arthrocentesis for him based on indications. After eight days of stay in the ICU, the patient's condition significantly improved with normalization of vital signs and improvement of cardiorespiratory status. The patient was discharged improved from the hospital in stable condition after a total of thirty days of intravenous antibiotics. The patient showed up on subsequent follow-up without any permanent cardiac sequel.

## 2. Discussion

Infective endocarditis (IE), first described in the mid-16th century, is a disease characterized by a high in-hospital mortality (17-30% mortality) [1–7]. IE is a microbial infection of the heart valves, intracardiac device, septal defects, mural endocardium, or rarely the subvalvular apparatus. Right-sided infective endocarditis (RSIE), IE of the tricuspid or pulmonic valve, is a rare entity accounting for 5-10% of all infective endocarditis cases. Most cases of RSIE (90%) involve the tricuspid valve [8–11]. The rarity of right-sided congenital heart diseases and rheumatic heart diseases coupled with the low pressure/low oxygen saturation on the right chambers of the heart is believed to be the reason for the lesser frequency of RSIE as compared to the left-side infective endocarditis [6, 11]. RSIE can be divided into three groups according to the underlying risk factors: intravenous drug users (IDUs), cardiac device carriers, and the “three noes” group (no left-sided IE, no IDUs, and no cardiac devices). Tricuspid valve endocarditis in nonintravenous drug users can occur in a variety of conditions including congenital heart disease, intracardiac devices, central venous catheters, and immunologically debilitated patients [5, 6, 9]. Intravenous drug use and the presence of cardiac devices (pacemakers, implantable automatic defibrillators, and central venous catheters) are the well-known risk factors for RSIE. Its occurrence in the absence of these risk factors and in otherwise normal heart is less understood. There are rare reports of RSIE following septic abortion, abscess, and septic arthritis. Globally, reports of tricuspid valve infective endocarditis (TVE) are growing because of the increasing frequency of intravenous drug use [5, 6].

Right-sided infective endocarditis usually presents with fever, persistent bacteremia, and septic emboli to the lungs. Initial presentation may comprise haemoptysis, cough, or chest pains which are related to septic emboli to the lungs. Pulmonary events occur in 80% of these cases and vary from minor atelectases to large infiltrates, pleural exudates, and cavitations. Septic shock, renal failure, and uncontrolled disseminated infection are common features of patients with RSIE without notable risk factors. Systemic emboli are rare manifestations of TVIE and, when noted, should be considered evidence of either left-sided involvement or paradoxical embolism. The peripheral stigmata of IE are not consistently present. Cardiac complications of TVE are much less common than those of left-sided endocarditis. The commonest cardiac complication of TVE is tricuspid regurgitation, the severity of which is a predictor of mortality besides vegetation size. Clinical suspicion of TVE should be raised in the presence of “tricuspid syndrome” characterized by recurrent pulmonary events, anemia, and microscopic hematuria [7, 9, 12].

Classically infective endocarditis is diagnosed using the modified Duke criteria, but the sensitivity and specificity of this criteria in right-sided endocarditis has not been studied; hence, a high index of suspicion is required for the diagnosis. A transesophageal echocardiography may be required in the detection of vegetations on the pulmonary valve and for the exclusion of left-sided valve involvement. The two most important diagnostic features of TVE are echocardiography evidence of vegetation and the presence of septic embolic phenomena. The vegetation of TVE is detected more commonly on the anterior leaflet of the valve and it is mostly large (>2 cm) due to low pressure, which enables the vegetation to grow large. Vegetation size correlates with mortality. TVE vegetations are highly mobile, which explains the higher rate of pulmonary embolic phenomenon associated with the entity [5–7, 10, 13].

*Staphylococcus aureus* is the commonest causative organism of isolated TVE followed by the *Candida* species, *Pseudomonas aeruginosa*, coagulase-negative *Staphylococcus*, *Corynebacterium* species, *Lactobacillus*, *Neisseria* species, and *Bacillus cereus* [9–11, 14, 15].

Medical therapy remains the mainstay of management for TVE. Intravenous antibiotics tailored to culture and sensitivity results with initial coverage of presumed organism are the mainstay of treatment. *Staphylococcus aureus*, being the commonest cause, should always be covered with effective antibiotics. In IVDUs with underlying valve lesions and/or concomitant left-sided involvement, coverage for streptococci and enterococci should be included. The duration of antibiotics depends on the following factors: the type of organism identified, vegetation size, presence of metastatic infections, involvement of left-sided valves, involvement of prosthetic valves, and immune status of the host [7, 13].

Surgical treatment is needed in 5-16% of TVE, and it has to be considered in the following situations: (1) intractable right-sided heart failure with poor response to diuretics, (2) persistent bacteremia despite the use of appropriate antimicrobial therapy, (3) large vegetation (>20 mm) that does not diminish in size despite repeated embolism, (4) fungal endocarditis, (5) concomitant left-sided IE, and (7) prostatic valve endocarditis. Valvectomy, valve replacement, or repair are the common surgeries performed for tricuspid valve endocarditis [7, 10, 13].

As compared to left-sided endocarditis, generally, the prognosis of TVE is more favorable and many patients can be cured with medical treatment alone. Overall mortality for TVE is between 5 and 15%, but could be as high as 30% in those lacking the traditional risk factors (intravenous drug use and cardiac devices) [7, 9, 13].

## 3. Conclusion

The symptoms of TVE are similar to those of respiratory infection (fever, dyspnea, and pulmonary infiltration), making misdiagnosis likely. Hence, TVE should be included in the differential diagnosis of patients presenting with febrile syndrome and pulmonary manifestations even in the absence of IDUs or cardiac devices.

## Data Availability

All important data are included in the manuscript.
